# Supplementary material for: Are carbon emissions trading and green financial instruments synergistic? -Comprehensive quantitative research based on content analysis
Source: PLoS One. 2024 Mar 7;19(3):e0298601. doi: 10.1371/journal.pone.0298601 (PMC10919601; doi:10.1371/journal.pone.0298601)
Supplement: S1 Data — (ZIP) [file pone.0298601.s001.zip › Data/地级市Z_F工作报告.zip!/╝¬┴╓/╦─╞╜╩╨/└┤╫╘╣1⁄2╓┌║┼í╛┬φ┐╦╩2╛▌═°í┐_2016.docx]

各位代表：

　　现在，我代表市人民政府，向大会报告政府工作，并对四平市“十三五”规划《纲要(草案)》作出说明，请予审议。

　　一、2015年工作回顾

　　2015年，是“十二五”规划的收官之年。一年来，在省委、省政府和市委的坚强领导下，在市人大、市政协的监督支持下，市政府紧紧依靠全市各族人民，纵深实施“一核三带”富民优先战略，主动克服经济下行压力，在破解难题中前行，在规范调整中进取，确保全市经济稳中有进、稳中向好。预计地区生产总值增长6.6%，一般公共预算收入增长7.7%，社会消费品零售总额增长10%。市七届人大三次会议确定的主要目标任务顺利完成。

　　过去的一年，我们重点做了以下工作：

　　——扩大投资拉动经济增长。务实推进项目建设。始终把投资作为稳增长的“定海神针”，推进项目谋划和开工建设。预计固定资产投资完成883亿元，增长15%。建设亿元以上项目255个，其中“4·18”集中开工34个。新增建设用地725公顷，增长122%。君乐宝乳业20万吨酸奶及乳酸菌饮料、君汇能源40万吨石油化工仓储及销售、绿舟科技LED光电照明产业化基地、玛钢7000万套曲轴、吉林天泽二氧化碳综合利用、大孤山景区生态旅游综合开发、银河中空玻璃及玻璃深加工等重大项目落地开工。佳乐宝日产250吨毛油精炼、宏宝莱10万吨饮品技术改造、昌源禽业日产10万只肉鸡屠宰加工技术改造、欧维姆年产3000台预应力建筑机械、公主岭市国文三甲医院迁建、绿健生物1000吨阿拉伯糖、双龙玻璃棉产业园、辽源—伊通—长春铁路等项目投产运营。大力开展招商引资。积极参加“民企吉林行”、“央企走进吉林”和东博会等招商活动，实施小分队定点精准招商，预计省外投资3000万元以上招商引资项目到位资金400亿元。建立招商引资基础数据库和重大项目跟踪推进机制，列入省招商库项目71个。预计实际利用外资3.4亿美元，增长12.5%。向上争取开发区发展引导资金2300万元。努力拓宽融资渠道。组建城市发展投资控股有限公司，与浦发银行、光大银行签订战略合作协议，与国开行、农发行、浦发银行、建设银行、交通银行、吉林银行等广泛合作，共融资到位资金25.17亿元，其中用于偿还债务及解决历史遗留问题11.7亿元，初步破解了城市基础设施建设融资难的问题。争取国家新增和置换债券到位资金10.75亿元。利用PPP模式融资3.56亿元。预计全市保险公司赔付金额8.5亿元，增长15%，充分发挥了经济补偿和风险管理功能。

　　——大力推进产业转型升级。创新驱动战略深入实施。预计实施技改项目275项，完成投资341.6亿元、增长13.2%；首次投产新产品202种，新产品产值率达13.9%。成立吉林省换热设备产业发展战略联盟，确定“THT”为换热器产业共享商标。康达农机与吉峰集团实现嫁接重组。百威英博金士百啤酒提质改造、2万吨线路金具嫁接改造等项目稳步推进。负压蒸汽冷凝器填补国内空白，免耕播种机、步枪专用工装模具等产品打入国际市场。创建全国质量强市示范城市进展顺利，荣获省质量奖企业达到6户。中国驰名商标新增5件，省名牌产品新增13件。国家可持续发展实验区通过中期验收，新建“吉林梨树黑土地保护与利用”院士工作站，吉林省医药中间体中试中心获批，新增国家和省科技企业11户、省级企业技术中心6户，列入国家和省科技发展计划项目52个，技术交易合同额1799.5万元。14个科研成果获省科学技术奖。工业经济稳步增长。预计工业总产值、增加值分别增长5.5%、7.5%。及时出台保增长促转型20条、精准调控19条等政策措施。设立产业引导资金5000万元，为53户工业企业提供资金支持。为企业提供担保贷款25亿元、争取专项资金3862万元、争取电费补贴1248万元、提供助保金池贷款8715万元。实现企业股权融资1.5亿元。艾斯克公司成功在新三板挂牌。在棚户区改造、城市道路、地下综合管廊等建设中采购地产品26.17亿元。市规划、国土、住建、消防等部门为115户企业解决规划、土地、产权等遗留问题。工业规上企业新增70户，达到559户。全市纳税超亿元企业5户，与上年持平；超千万元企业87户，增加14户；超百万元企业517户，增加51户。民营经济发展活跃。创建国家级中小企业公共服务示范平台1个、省级16个。新增个体工商户18934户，增长5.23%；新增私营企业3518户，增长18.75%。民营经济主营业务收入完成2745亿元，增长9%。服务业不断壮大。电子商务产业园区被商务部批准为“国家电子商务示范基地”。公主岭市中国玉米云商城上线企业达到502户。伊通满族自治县被财政部、商务部批准为电子商务进农村综合示范县。亿赞普“中国－东北亚跨境电子商务平台”正式上线，“三农红E邮”等农村电商服务站成立运营。万达购物广场进入内装阶段，黄金珠宝交易中心启动招商，万嘉购物广场进展顺利。加强旅游景区基础设施建设，满族民俗游、乡村休闲游、红色旅游加快发展。

　　——全面发展农村经济。“三区一建”成效显著。建设粮食高产示范区100个，示范面积108万亩，建成高标准基本农田51万亩。农业科技贡献率达到58%，新建气象标准化作业站点34个。有效应对较重旱灾等自然灾害，预计全市粮食总产150亿斤。新建扩建棚室蔬菜园区25个，新增棚室面积5315亩。新建扩建牧业小区209个。投入农村人居环境建设专项补助资金4122万元，创建省级以上美丽乡村10个、市级美丽乡村25个，打造精品线路12条，评选美丽庭院1万个、干净人家3万户，全省新农村建设现场会在我市召开。农业产业化规模壮大。全市农产品加工业实现产值556.5亿元，增长16.9%。温氏100万头猪养殖、新希望农畜科技产业园等项目开工建设。投资8.1亿元新建扩建省级农业产业化项目15个，4户企业通过省级农业产业化重点龙头企业评选。梨树县喇嘛甸镇王家园子村被命名为全国“一村一品”示范村。双辽一马树森林公园等8家省休闲乡村旅游星级企业通过复评，梨树北方巴厘岛等农业休闲项目开发升级。农民合作社发展到12694个，增长33.58%；家庭农场发展到1857个，增长52%。品牌农业创新发展。出台“三品一标”农产品认证办法，新增“三品一标”产品58个。天成玉米公司玉米油被农业部认定为绿色食品。“双辽小米”、“双辽花生”、“伊通大米”被工商总局认定为地理标志证明商标。农业机械化水平持续提高。争取各类农机补贴2.76亿元，购置各类农机具9558台，受益农户7983户，带动农机销售9亿元。农机总动力达到350万千瓦，增长13.6%；耕种收综合机械化水平达到78%，提高3个百分点。

　　——规范整治城市建设管理。规划引领约束作用明显增强。完成四梨同城化区域发展战略规划及核心区总体规划，编制实施中心城区地下综合管廊和棚户区改造规划。严格规划审批程序，实现规划一张图、审批“四支笔”。出台规划监察管理办法，加大巡查执法力度，全力遏制违建增量，减少违建存量，查处违建338处、75203平方米。改造与新建整体推进。积极与央企合作，建成紫气大路、北迎宾街等4个地下综合管廊项目8.98公里。新建续建市区道路27条、18.87公里。实施市区主要交通路口渠化和交通信号灯升级改造。完成供热管网82公里、燃气管网35公里、雨水污水管网38公里改造。四平污水处理厂、梨树污水处理厂升级改造工程全面完工，郭家店污水处理厂、叶赫污水处理厂开工建设，全市4个出省境河流断面水质明显好转。完成10条街路绿化面积14万平方米，城区绿化覆盖率达到37.9%。全市造林绿化25万亩，森林覆盖率达到19.1%，清收林地87.9万亩。规划展示馆基本完成布展，图书馆、体育馆开工建设，千亩植物园建设积极推进。城市管理不断规范。采购洗扫车64台，市区街路机械化清扫率由17%提升到85%。建立严格的房地产市场准入机制，禁止侵占校园及影响周边环境的开发行为。下大气力解决棚户区改造和公租房建设中存在的问题，完成71万平方米历年拖欠的开工建设任务。顶住重重压力，解决三达、盛世华庭、草拉房、6＃-1-3等棚户区改造遗留问题，全力保障回迁户利益。平安城市、智能交通、智慧安监、智慧食药监等智慧城市项目顺利推进，一卡通、智慧城管项目启动实施。交通建设成效显著。长平高速八车道改造、长双高速大岭至双辽段竣工通车。新建改建农村公路216.3公里。军民合用机场项目已进入国家审批快车道。

　　——扎实推进全面深化改革。建立政府部门权力清单，清理后保留部门职权1883项，精简50.2%，非行政许可事项实现“零审批”。加快商事制度改革，实施“五证合一、一照一码”登记制度，在全国市辖区率先推行“一个工商窗口发执照”，在全国地级市率先建立工商登记“负面清单”。完成食品药品质量安全监管机构改革。县级公立医院综合改革全面启动。推进职业教育体制改革，做大做强高等职业教育，市商业学校和市轻化工学校并入四平职业大学。制定市直行政事业单位、市属企业国有资产管理规定，全面清理登记和规范管理国有资产。出台土地征收补偿安置办法，依法规范土地征收。完成不动产统一登记职责和机构整合。深入推进供销社综合改革试点，启动组建供销集团。3家“粮食银行”企业挂牌运行。确立胜利乡、卧虎镇、马鞍山镇为集体产权交易中心改革试点。范家屯镇、孤家子镇、郭家店镇被列为全省扩权强镇试点。深入开展农村土地确权登记试点，公主岭市整体推进工作进入颁证阶段。全市土地收益保证贷款达到8亿元。梨树县被确定为全国农村金融综合改革试验区。

　　——保持社会大局和谐稳定。国家安全生产监管防控体系试点建设全面推进。出台安全生产监管执法实施意见，安全生产隐患排查治理体系上线运行，安全生产专项行动常态化，全年未发生较大以上安全生产事故。推进信访工作规范化，化解信访积案146件，依法严厉打击非访缠访闹访行为，进京非访、去省集体访量分别下降81.3%、37.2%。完善人民调解组织体系，调解矛盾纠纷2.8万件。举办纪念“宪法日”等法治宣传教育活动，“六五”普法通过省验收。建立刑事警情每日研判制度，命案破案率100%。落实24小时巡逻防控机制，集中整治治安复杂区域，开展交通、消防及网络整治行动，社会治安形势持续好转，人民群众的安全感满意度不断提升。

　　——切实保障和改善民生。市区民生支出57.1亿元，占本级财政支出的78.4%，年初确定的35件民生实事基本完成。预计城镇和农村常住居民人均可支配收入分别增长6.5%、7%。扶持成立创客创业园，发放创业小额贷款6100万元，农村劳动力转移就业47万人，全市新增就业4.15万人，城镇登记失业率控制在3.68%以内。提高城乡居民养老保险待遇，基本养老金足额发放。城镇职工和城镇居民医疗保险报销水平较上年提高10%，在全省率先做实城镇居民大病补充保险。启动事业单位工伤保险，在全省率先下放医保转诊审批权，实现省内异地就医即时结算。城市低保月标准提高到444元，农村低保年标准提高到2684元，五保集中和分散供养标准分别提高到4500元、3000元。全面建立临时救助制度，（上接一版）重特大医疗救助封顶线达到2万元，提高66%。市社会福利院改造工程主体封闭，城市社区老年人日间照料中心实现全覆盖。棚户区改造开工40.59万平方米，新建公租房24.56万平方米，暖房子改造120万平方米。整治弃管小区30个，指导成立业主委员会22个。新增公交线路2条、公交车8台。淘汰燃煤小锅炉121台，减少燃煤量12.5万吨。完成4.13万户二次供水工程改建任务，农用沼气、农垦危房改造工程通过验收。完成71处农村安全饮水工程，解决了4.85万农民和1.03万师生饮水不安全问题。创建2个食品安全示范县（区），完成2476户“四品一械”可追溯体系终端安装并使用，改造阳光厨房125户，建设放心粮油示范店20家。新建农村公办幼儿园15所，美丽乡村学校达标40所，四平盲童学校综合楼竣工，市中小学社会实践教育中心主体封闭，铁西区、铁东区和伊通满族自治县通过国家义务教育基本均衡发展评估验收。建成标准化村卫生室234个，创建国家中医药工作先进单位通过验收。开展文化“七进”、“三下乡”活动，完成四平战役纪念馆陈列改造，改编吉剧《温莎》剧本获“田汉戏剧奖”一等奖，并晋京成功展演。大力开展全民健身活动，成功举办社区体育大赛、第五届市直机关职工运动会，城区社区和行政村健身器材全覆盖。参加省青少年各项比赛获金牌55枚，列全省第二位。吉林师范大学网羽中心建成并投入使用。

　　——不断加强政府自身建设。深入开展“三严三实”专题教育，突出规范管理，加强制度建设，以更严标准更实作风推进政府工作。严格执行重大决策程序，市政府常务会议出台政策措施29件。发挥市政府法律顾问团作用，受理城市建设、经济补偿、产权权属、代理诉讼等涉法事务89件，办结80件。机构编制一律经编委会例会研究，取消审批“一支笔”。建立39个政府部门重点工作任务落实管理系统，强化考核、调度和督查。办理人大代表建议意见96件、政协委员提案189件，办复率100%。完成全流程行政审批系统升级改造，政务大厅审批即办件率达到91.8%。规范财经秩序，“三公经费”下降4.5%。加强审计监督，完成审计63项，振兴东北老工业基地项目跟踪审计56项。加强行政效能、执法、廉政监察，共查办案件821件，给予党政纪处分869人，移送司法机关29人。

　　妇女儿童、民族宗教、广播电视、知识产权、外事侨务、人民防空、地震监测、通信、档案、老龄、地方志、残疾人等事业不断进步。

　　各位代表，过去一年四平取得的成绩，是市委总览全局、科学决策的结果，是市人大、市政协有效监督、大力支持的结果，是全市人民同心协力、艰苦奋斗的结果。在此，我代表市政府，向全市人民，向全市人大代表、政协委员，向全市各民主党派、工商联、人民团体，向全市政法干警、驻平人民解放军及武警官兵，向驻平中省直各单位和所有关心支持四平发展的国内外朋友，表示崇高的敬意和衷心的感谢！

　　我们也清醒地看到，四平振兴发展还存在不少困难和问题，稳中有忧、进中有难。一是经济总量不大、质量效益不高、创新能力不强、产业结构不优、服务业发展滞后等问题尚未根本解决。二是稳增长调结构压力增大，少数企业处于停产半停产状态，项目谋划和建设力度不够，缺少立市立县的大项目、税源型项目，财政收支矛盾仍很突出。三是资源环境承载压力加大，大气污染防治形势严峻，垃圾污水处理能力有待提高，生态文明建设任重道远。四是城市服务管理水平不高，老城区基础设施欠账较多，历史遗留问题破解难、征地拆迁难等问题依然存在，房地产开发市场混乱，百姓回迁难回迁慢，露天烧烤、占道经营、乱贴乱画等痼疾顽症久治不愈。五是少数干部精神萎靡不振、工作状态不佳，慵懒散、推躲拖等现象严重，不作为、慢作为及不愿担当、不敢担当、不会担当问题比较突出。个别干部在严肃的纪律面前仍不收手、不收敛，“四风”和腐败等违规违纪违法问题还时有发生。我们必须直面和正视这些困难问题,不麻木、不回避、不绕弯,以更大的决心和勇气积极应对、有效解决。

　　二、2016年工作安排

　　今年是实施“十三五”规划的开局之年，也是市委提出的两年“夯实基础”的第一年，发展任务繁重艰巨，外部环境复杂多变。面对新形势、新要求、新使命、新期待，市政府将牢固树立创新、协调、绿色、开放、共享的发展理念，按照市委的统一部署，破解发展难题，增强发展动力，夯实发展基础，厚植发展优势，只争朝夕，埋头苦干，努力实现全面建成小康社会决胜阶段的良好开局。

　　今年政府工作的总体要求是：全面贯彻落实党的十八大、十八届三中、四中、五中全会精神和省委十届六次全会精神，按照市委六届六次全会和经济工作会议部署，以创新转型、结构调整为主线，纵深实施“一核三带”富民优先战略，紧紧围绕稳增长、调结构、惠民生、防风险，强力推进“五路并进”、“五市联动”，打好“六个攻坚战”，为完成“十三五”规划目标任务、打造“美丽四平、幸福家园”奠定坚实基础。

　　主要预期目标是：地区生产总值增长7%，一般公共预算收入增长7%，工业增加值增长5.5%，社会消费品零售总额增长9.5%，城镇和农村常住居民人均可支配收入均增长8%，居民消费价格指数涨幅控制在3.5%以内，调查失业率控制在6%以内，万元GDP能耗下降3.2%。

　　围绕上述目标任务，重点做好以下工作：

　　（一）发挥投资关键作用，保持经济稳定增长。加快重点项目建设。扩大有效投资，优化投资结构，加大对基础设施、科技创新、生态建设、民生改善等领域投资。固定资产投资计划增长12%。组织实施一批补短板、上水平、增税源、有后劲的重大项目，建设亿元以上项目278个，其中春季集中开工70个以上。重点抓好现代农机装备区域创新示范基地、颐高国际电子商务产业园、大清鹿苑产业园、生态化工产业园、新盛科技玻璃深加工产业园、君乐宝牧场、天成现代物流、中国农业大学科技孵化园、中部城市引松供水工程等项目。推进梨树绿色食品产业园、大孤山景区生态旅游综合开发等项目建设进度。促进君乐宝乳业20万吨酸奶及乳酸菌饮料、线路器材厂产业转型升级改造、年产3000台大型联合收割机、吉林天泽二氧化碳综合利用、中心医院内科大楼、黄金珠宝交易中心等项目投产运营。强化项目谋划，落实项目包保，建立联席会议制度及在建项目问题清单、责任清单，认真核实开工项目，抓紧落实建设条件，超前夯实前期工作，推进项目逐月滚动开工建设。加大招商引资力度。组织参加“台资企业吉林行”、“世界500强企业走进吉林”等活动，围绕支柱产业、优势产业、新兴产业和现代服务业开展对接招商；发挥开发区、工业集中区、特色园区“火车头”作用进行务实招商；组织小分队与重点区域、央企、民企和商协会进行点对点精准招商。建立招商项目谋划联动、市级领导包保、跟踪调度和绩效考核等机制，推进更多更好项目真正落地。进一步拓宽融资渠道。赋予城市发展投资控股有限公司投资运营国有资产职能，加大国有资产整合力度，充分发挥国有融资平台作用，与国家政策性银行、商业银行、股份制银行开展深入合作，不断扩大融资投资规模。推进国有资产证券化和PPP合作模式，规范发展融资租赁、互联网金融、产业基金、股权投资等新业态。积极向上争取专项资金，力争完成项目专项资金增长50%以上。

　　（二）推动产业转型升级，提升产业竞争力。立足“工业强市”，挺起四平经济发展脊梁。加快调整产业结构。实施主导产业振兴工程。围绕装备制造业，推进换热设备工艺装备升级试点，发挥换热设备产业战略联盟作用，加快10万平方米负压蒸汽冷凝器等项目建设，创建国家换热设备产业集群品牌示范市。推进与中国农机工业协会、中联重科、吉峰集团、罗斯托夫农机集团合作，建设大型农机生产线，推进大马力联合收割机组装生产线落地，支持免耕播种、秸秆打包、粉碎压实等农机提质扩能。加快专用车结构调整，建设省级特种车研发中心，大力发展环卫、冷藏、工程、消防、物流等特种车，开发房车、旅游景点观光车，谋划建设国家旅游装备制造产业园区。围绕农产品加工业，扩大调整烟酒食糖规模结构，延伸拓展畜禽食品、绿色饮品和粮食生化三大链条，推进烟厂规模、结构双提升。编制四平市绿色食品加工园区规划，加快建设梨树农畜产品加工园区。围绕能源产业，推进油气田项目建设，积极勘探开发地热能资源。建设风力发电基地、生物质利用基地和太阳能发电示范区。争取国家新能源汽车试点城市，制定新能源汽车运营发展规划，大力发展新能源汽车。实施优势产业壮大工程。加快医药健康产业发展，发挥吉春制药院士工作站作用，壮大梅花鹿保健品产业，谋划研究开发“满药”。加快化工产业园建设进程，提升精细化工、日用化工能力水平，推进基础化工原料产业转型发展。实施传统产业提升工程。大力发展绿色建材产品，提升玻璃产业集群集聚水平，综合利用废渣生产高标号特种水泥及制品。推进创新驱动战略。瞄准“中国制造2025”，加快企业嫁接改造，推动换热器国检中心事业转企业改革，建立名牌产品数据库，完成创建全国质量强市示范城市考核验收。深入开展“院士专家四平行”活动。加大人才引进力度，从今年开始，“十三五”期间每年从985、211工程重点高校引进100名左右全日制硕士、博士人才。加强与长春应化所、长春光机所、吉林大学、长春理工大学、吉林师范大学等院校合作，打造10个拥有核心技术和自主品牌高新技术产品，培育实施50个高新技术项目，建成科技大市场并投入使用。支持推进“四平创造”工程，启动企业技改滚动计划，实施技术改造项目177项，完成投资346亿元。着力培育新的增长点。完善民营经济和中小企业服务体系，培育新型创业孵化器，设立创业投资引导基金，吸引天使投资等创业投资，新增民营企业1000家、个体工商业户8000户。完善扶持企业政策措施和领导包保产业机制。推进供给侧结构性改革，改变传统产品占大头、“原”字号“初”字号产品居多的单一产品结构。新增规上工业企业50户以上。加强企业家队伍建设，支持成立企业家民间商会，增强企业家发展壮大的信心和回报四平人民的热情，打造一支依法经营、照章纳税、尽社会责任的四平企业家精英团队。在全社会营造尊重、扶持、爱护、感恩企业家的浓厚氛围，鼓励与企业家交朋友，反对与企业家搞交易，打造积极健康、富有正能量的政商关系。

　　（三）注重补短板优结构，发展现代服务业。优先发展生产性服务业。大力发展现代物流业。构建现代物流产业体系，加快推进豪德商贸物流、吉高多式联运货运枢纽、浙江新农农副产品商贸城、内陆港等项目建设。全面发展金融服务业。谋划建设四平金融街，打造金融超市，引进和集聚各类金融服务机构。发展普惠金融，加强对中小微企业和农村的金融服务，完善融资担保体系，做强助保金池，鼓励银行、保险、担保、典当、融资租赁等机构开发融资产品。加快发展信息服务业。大力推进“互联网+”行动计划，谋划建立全域数据处理应用中心和集中信息资源库，整合智慧城市与信息惠民平台资源，打造智慧城市。加快建设电子商务产业园、放心粮油电子商务平台、大宗农产品交易中心等项目。深化与亿赞普等大型跨境电商企业合作，推进“中国－东北亚跨境电子商务平台”建设，扩大对外出口。发展科技研究、工业设计、信息咨询、技术培训等行业，支持企业建立检测中心、工程实验室，推动企业由生产型向服务型转变。着力提升生活性服务业。围绕“商旅兴市”，全力创建“旅游城”。编制《四平市全域旅游发展总体规划》，加快推进国家旅游商品（纪念品）集散中心、东方美食城、旅游休闲康乐城、君汇集团休闲观光园、玄武湖旅游创新与科技示范区等项目建设，着力发展红色文化、工业文化、萨满文化和休闲观光旅游。加快发展养老服务业。发展居家养老便捷服务，按照居家老年人的需求提供定制服务。打造“生态养生”、“休闲度假”等特色养老服务产业园区。提升商贸流通业。加快传统商贸服务业改造升级步伐。推进万达城市综合体、欧亚城市综合体、万嘉购物广场等项目建设，不断提升商业业态层次。

　　（四）加快县域突破，推进农业现代化。围绕城镇化建设，加快县域经济发展。依托产业基础和资源禀赋，积极探索符合县域实际的发展道路，宜工则工、宜农则农、宜商则商、宜游则游。按照“有中生新、无中生有”要求，做大做强主导产业，培育壮大优势产业，谋划生成新兴产业，逐步形成支撑县域经济发展的产业体系，为县域突破奠定基础。推动实施新型城镇化规划，加快四梨同城化进程，推进梨树县国家新型城镇化综合试点，提升县城和重点小城镇产业功能、服务功能和居住功能。加快孤家子、郭家店、营城子、茂林、叶赫等节点镇建设，围绕长平经济走廊打造12个特色城镇。围绕“农业立市”，推进“三个体系”建设。加快建立现代农业产业体系。以提高单位土地面积收益为导向，调整农作物种植结构，开展粮食绿色增产增收攻关活动，完成10万亩以上玉米种植调减任务。发展特色高效园艺特产业，新建棚室蔬菜园区10个，新增面积2000亩；改造提升棚室蔬菜园区11个，改造面积3000亩。深入开展畜禽养殖标准化示范创建活动，新建改建标准化牧业小区150个。加快建设现代农业生产体系。推进主要粮食作物生产全程机械化，耕种收综合机械化水平达到80%以上。启动实施四平市黑土地保护治理工程，集中连片建设高标准农田，加快建设农业物联网试点项目。高质量完成第三次全国农业普查工作。加快建立现代农业经营体系。大力培育家庭农场、农民合作社、龙头企业等新型农业经营主体，开展示范家庭农场和农民专业合作社示范社创建行动。支持成立四平市农业产业商会。新增“三品一标”产品认证40个。梨树县、铁西区整建制全面启动农业现代化建设。围绕环境整治，加快新农村建设。编制县域村镇体系规划，发展农村新型社区，推进城郊村整体搬迁进城、空心村向中心村集中。巩固提高303线，强力推进102线，全域提升市辖区农村环境，打造13个精品示范片区。强化产业布局，提升文化包装，注重景观建设，持续开展农村环境清洁工程和美丽庭院、干净人家评选活动。加快环境改观、住房改善、厨房改良、厕所改造、生活习惯改变，全面改善农村生产生活条件。

　　（五）加强生态环境保护，加快宜居城市建设。综合整治人居环境。围绕“生态育市”，市区建成高污染燃料禁燃区15.6平方公里，从源头上控制大气污染，完成省政府下达的总量减排任务。修订实施大气重污染应急预案，继续开展工业污染防治专项行动，严格机动车尾气减排管理，完成天成玉米生产废气收集处理工程。研究推进农业秸秆综合利用，整治秸秆露天焚烧，减少大气污染。划定生态保护红线，谋划实施辽河流域（四平段）综合治理、伊通河治理工程。启动实施“海绵城市”建设。推进西湖湿地建设，完成南北河、郭家店、叶赫4个污水处理厂建设，启动一水厂扩建、城市防洪北河治理、转山湖水库引水工程。完成街路、广场、庭院、小区以及南北河沿岸等绿化提升任务，市区义务植树5万株。加大清收林地还林力度，恢复建设农田防护林2400亩。造林绿化25万亩。争取耕地轮作休耕制度试点。加快基础设施建设。完成续建地下综合管廊9条、10.99公里，新建地下综合管廊7条、10.36公里。城区新建道路18条、13.22公里，续建道路3条、5.48公里。博物馆、大剧院、南环城市公交保障中心开工建设，图书馆、体育馆完成主体工程，东客运枢纽投入使用，规划展示馆和千亩植物园正式对外开放。开工建设集双高速公路四平段、伊通至长春国道一级公路伊通段、国道绥沈二级公路长岭至双辽段、二龙湖旅游公路叶赫至杨木林段，续建国道牙克石至四平一级公路梨桑段，实施省道长春至双辽二级公路养护工程、长平高速四平出口景观绿化提升工程。组建四平军民合用机场有限责任公司，力争机场项目年内开工建设。严格规范城市管理。开展城区环境综合整治百日会战，确保通过国家卫生城复检验收。实施最严格的房地产准入制度，吸引优质诚信开发企业进入四平，严禁侵占绿地、校园及影响周边环境的开发行为，将进入“黑名单”的开发企业一律清除四平房地产市场。加大棚户区改造、房地产开发遗留问题的解决力度，确保动迁群众尽快回迁、妥善安置。加大规划监察力度，以“零容忍”的态度，依法拆除违法违章建筑，坚决遏制违建增量，全面减少违建存量。加大土地征管力度，依法处置批而未供、供而未用等闲置土地。以更大的决心坚决斩断伸向拆迁和征收补偿的幕后“黑手”。加大交通管理力度，依法严厉处罚各类违法违规行为，确保交通安全畅通。充实完善城市管理委员会，组建综合执法机构，解决露天烧烤、占道经营、乱贴乱画、乱钉地桩地锁等顽症，让城市居民生活更方便、更舒心、更美好。

　　（六）坚持体制机制创新，推进全面深化改革。推进简政放权。坚持放管结合，持续优化服务，该放的事项全部放到位，该管的事项管住管好。加快完善政府权力清单、责任清单，划定权责边界。深化行政审批制度改革，规范审批行为和中介服务，加强事中事后监管，完善政务大厅重大项目并联审批和直通车制度，所有应进大厅审批项目必须无条件进入。推进商事制度改革，出台市场主体信用信息管理办法。深化食品药品监管体制改革。完善国有资产管理体制。摸清国有资产底数，严格国有资产处置程序，盘活用好国有资产，确保国有资产保值增值。完善现代企业制度，健全法人治理结构，严格监管国有控股投资公司，规范国有资本投资运营。加快专项领域改革。完善预算管理制度，推进市对区、开发区财政管理体制改革，调动区和开发区当家理财的积极性。依法规范投融资平台建设，支持企业上市、发行债券，降低融资成本。继续拓展农村土地承包经营权确权登记颁证试点、农村土地经营权抵押贷款、承包收益保证贷款和林权、水权、草权等物权融资试点。抓好梨树县全国农村金融综合改革试验区工作。加快辽河垦区行政体制改革步伐。推广“粮食银行”试点经验。实施县级供销社综合改革试点。启动国有林场改革。组建四平水务集团。

　　（七）强化治理能力建设，全力构建和谐社会。创新社会治理模式。推进政府从管控规制转向法治保障，从运用行政手段转向综合运用多种手段。加强基层社会治理，培育发展社会组织，增强社区服务功能，倡导社会志愿服务。推进法律援助中心机制转型试点。切实抓好安全生产。纵深推进“四化融合”、“三位一体”安全监管防控体系、国家安全生产综合改革试点和全国安全生产隐患排查治理体系建设，制定安全生产事故隐患举报奖励实施细则，彻查和整治安全隐患，坚决防止重特大事故发生。压实信访维稳责任。完善信访工作机制，发挥联席会议作用，建立联合接访模式，限期办结初期访案件，依法解决非访问题，强化信访考评问责，推进信访形势进一步好转。深化平安四平建设。完善社会治安防控体系，提升反恐处突能力，加大打击违法犯罪力度，保持命案侦破率100%。强化巡逻机制，市区视频监控实现全覆盖、无盲点，进一步提升群众的安全感和满意度。

　　（八）突出保障改善民生，实现发展成果共享。推动更高质量就业。开展大众创业、万众创新系列活动，筹建公共实训基地和创业创新平台，城镇新增就业3.4万人，农村劳动力转移就业35万人。增强社会保障能力。推进保险征缴扩面，启动机关事业单位养老保险制度改革，提高职工医疗保险待遇水平，提高中医诊疗、药品医保报销标准。完善城乡社会救助体系，全面核查城乡低保对象，提高城乡低保标准和五保供养标准，支持发展民办养老机构，推进政府购买养老服务试点，支持残疾人和慈善事业发展。深入开展双拥活动，实现全国双拥模范城“七连冠”。打响脱贫攻坚战。切实摸清底数，建档立卡，强化包保帮扶，明确落实责任，健全督查制度，严格检查考核，做到精准扶贫、精准脱贫，确保完成年度3万人脱贫任务。发展各项社会事业。围绕“文化铸市”，努力创建“文明城”，全面提升市民素质。坚持文化“六馆”优质开放，深入开展文化“七进”，举办首届英雄城艺术节。确保梨树县、双辽市义务教育基本均衡发展通过国家评估验收。继续整合职业教育资源，推进农村中小学规划布局调整，坚持立德树人，实施中小学“明德知礼”工程。做好成立四平市长影艺术学校前期工作。围绕创建“健康城”，落实改善医疗服务行动计划，启动建设四平市结核病防治所项目，实施全面两孩政策。广泛开展全民健身活动，加大体育器材投放力度，筹备建设滑冰馆，开工建设伊通国信南山滑雪场。组队参加全国第十三届冬运会，承办全国中等城市柔道邀请赛。

　　在全面做好各项民生工作的同时，重点完成30件民生实事。（1）市区棚户区改造37.06万平方米、5633套；（2）市区建成公租房11.64万平方米、2461套；（3）市区完成暖房子改造和提标31万平方米；（4）市区新增热源能力100万平方米，改造供热管网80公里；（5）市区淘汰燃煤小锅炉120台以上；（6）开工建设南湖高架桥、东丰立交桥、北仁兴街公铁立交桥；（7）市区改造人行步道10条、6.7万平方米；（8）完成仁兴商业街改造，全面取缔街面摊点；（9）完成市区亮化改造升级工程，环路及四梨大街新安装路灯1496组，主街路进行楼形灯装饰和路灯改造，巷路路灯全覆盖，“黑楼道”问题彻底解决；（10）市区新增公交线路10条，增加新能源电动公交车38台；（11）市区改造16条公交线路137个候车廊、462个停靠点；（12）完成南湖公园改造升级，建成休闲开放式公园；（13）市区完善40个弃管小区配套设施，维修巷路25条，实现物业管理20个；（14）市区新建垃圾转运站7座；（15）全面规范整治城区废旧物品收购点，还市民健康整洁生活环境；（16）新建改建农村公路200公里以上；（17）失业再就业8000人，困难人员就业4500人，解决城镇零就业家庭比率100%；（18）市、县最低工资标准分别提高到1380元、1280元；（19）职工医疗保险基本段封顶线由6万元提高到8万元，大额补充医疗保险封顶线由10万元提高到18万元；（20）提高农村低保标准，确保达到3100元以上的脱贫标准；（21）完成“红色舞台”演出15场、“红色经典影片展映”50场、送戏下乡演出70场；（22）改扩建农村公办幼儿园76所；（23）新建中医馆10个，实现乡镇卫生院中医科（中药房）全覆盖；（24）为1670户计划生育特殊家庭免费体检并发放特别扶助金；（25）为40个行政村新建室外体育设施，为城区20个社区更换体育器材；（26）为2000户食品药品生产经营企业免费安装可追溯体系终端设备，为300户餐饮服务企业免费安装油水分离器，新建食品药品便民服务站19个；（27）市区建设粮油配送中心2个、放心粮油示范店20家；（28）为全市群众免费提供法律援助2000件以上；（29）建成网上公安局，为市民在线咨询、行政审批和警务服务提供便利；（30）建设平安四平宣传教育中心，年内向市民免费开放。

　　（九）体现担当和从严，加强政府自身建设。坚持求真务实。始终坚持问题导向，突出解决各种问题。在解难题办实事上出硬招，在打基础利长远上下真功。不吹气注水说空话，不搞假把式练虚功。敢于较真较准较实，善于落细落小落地。搞清问题对症下药，持续跟踪确保成效。努力实现有质量、有效益、可持续的增长，不断创造经得起实践、群众和历史检验的业绩。严格依法行政。自觉接受人大、政协监督，认真办理人大代表建议意见和政协委员提案。实施《四平市法治政府建设规划（2016－2020年）》，建立政府立法机制，发挥政府法律顾问团、专家咨询团作用。推进政府信息公开。启动“七五”普法宣传教育。保障律师执业权利，规范律师执业行为。突出稳增长政策、政府重大项目的跟踪审计和民生重点专项资金审计，实现审计全覆盖。强化责任担当。增强忧患意识、创新意识、宗旨意识和使命意识，整治慵懒散、推躲拖行为，真正做到敢于担当、善于担当、体现担当。落实改革发展举措，履行民生改善职责，扛起“六个攻坚战”重任。高度重视破解千难万难，主动作为闯出大路小路，实现重点工作新突破。面对困难不退缩，遇到挫折不灰心，出现干扰不畏惧，鼓励改革创新，宽容挫折失误，严惩诬告陷害，真正形成干事创业的良好政治生态。全面从严治政。巩固“三严三实”教育成果，做到为民用权、为民干事、为民谋利。严格绩效考核，启动行政问责，大力整治不作为、慢作为、虚作为，严厉查处乱作为。加强政府廉政建设，严格执行《廉洁自律准则》和《纪律处分条例》，落实主体责任，履行“一岗双责”，始终把纪律挺在前面，不越红线，守住底线，持之以恒纠治“四风”，态度坚决惩治腐败，努力建设为民务实高效清廉政府。

　　三、关于《四平市国民经济和社会发展第十三个五年规划纲要（草案）》的说明

　　按照市委六届六次全会通过的《关于制定四平市国民经济和社会发展第十三个五年规划的建议》，市政府组织编写了《四平市国民经济和社会发展第十三个五年规划纲要（草案）》，已提请大会审议。

　　（一）“十三五”《纲要（草案）》编制过程。市委、市政府高度重视“十三五”规划编制工作，专门成立领导小组，深入开展调研座谈，广泛听取专家意见，多次召开专题会议，对事关四平发展的重大问题进行研究论证。从2014年4月开始，组织编制《“十三五”规划前期重大问题研究指南》，正式启动规划编制工作。先后召开各类座谈会50余次，收集社会各界意见1200余条。2015年7月，市人大常委会对“十二五”规划实施情况开展专题视察，并审议调整了“十二五”规划部分主要指标。10月末形成“十三五”规划基本思路。12月中旬按照市委六届六次全会《建议》，编制形成《纲要（征求意见稿）》，广泛征求各方面意见，并先后两次召开专家咨询论证会，对“十三五”规划纲要及重点项目进行咨询论证。之后，市政府召开常务会议专题讨论，并提交市委常委会议原则通过，最终形成提请本次会议审议的《纲要(草案)》。

　　（二）“十二五”时期经济社会发展取得的成绩。“十二五”时期，在省委、省政府和市委的坚强领导下，全市上下紧紧围绕“一核三带”富民优先战略，攻坚克难，埋头苦干，保持了经济社会平稳健康发展，综合实力显著增强。预计全市地区生产总值年均增长10%以上，一般公共预算收入年均增长17.4%，固定资产投资年均增长18.3%。累计建设亿元以上项目800余个，烟厂易地改造、迎新玻璃、巨元瀚洋新型能源换热器、一汽四平专用车年产6000辆轻金属桁架厢式半挂车、艾斯克禽类自动屠宰掏膛生产线、九洲光电LED-PSS基板生产线、万达城市综合体等一批重大项目相继落地。同时，全市创新转型稳步推进，深化改革打开局面，现代农业步伐加快，城乡面貌明显改观，民生福祉持续改善，较好完成了“十二五”规划主要目标任务。

　　（三）“十三五”《纲要（草案）》的指导思想和主要目标。指导思想是：高举中国特色社会主义伟大旗帜，以马克思列宁主义、毛泽东思想、邓小平理论、“三个代表”重要思想和科学发展观为指导，全面贯彻党的十八大和十八届三中、四中、五中全会精神，深入落实习近平总书记系列重要讲话精神和视察吉林重要讲话精神、省委十届六次全会精神，按照“四个全面”战略布局，遵循“五大发展”理念，适应经济新常态，以创新转型、结构调整为主线，纵深实施“一核三带”富民优先战略，坚持产业跃升、城乡统筹、生态文明、开放融合、民生改善“五路并进”，推进农业立市、工业强市、商旅兴市、生态育市、文化铸市“五市联动”，提升产业竞争力、城市承载力、社会影响力，确保如期全面建成小康社会，全力打造“美丽四平、幸福家园”。

　　发展目标是：确保如期全面建成小康社会，确保地区生产总值到2020年比2010年翻一番，城乡居民收入比2010年翻一番，到2018年末，现行标准下贫困人口全部脱贫。具体目标包括经济发展、社会民生、结构调整、改革开放、生态文明等五大方面27个指标。初步提出我市“十三五”时期GDP年均增长7.5%左右。主要基于四个方面考虑：一是确保实现GDP翻番目标，二是为转方式和调结构留出足够空间，三是要实现全省中等偏上的发展水平，四是综合考虑了要素影响和三次产业发展的支撑作用。

　　（四）“十三五”时期经济社会发展的主要任务。《纲要(草案)》共设置十五章57节。第一章阐述“十二五”时期经济社会发展主要情况，分析“十三五”发展环境，提出“十三五”发展总体要求。第二章至第十四章从13个方面提出全市经济社会发展的战略任务措施，主要是实施创新驱动战略，激活发展内生动力；转变农业发展方式，率先实现农业现代化；推动新型工业化，打造产业发展新高地；发展现代服务业，引领结构优化升级；实施全域信息化，建设网络强市；优化空间布局，加快区域协调发展；完善现代基础设施，增强发展支撑能力；强化绿色发展，建设美丽四平；拓展开放合作领域，开创协同发展新局面；完善发展新体制，释放发展活力；推进治理能力，构建包容和谐社会；加强精神文明建设，打造文化名市；保障改善民生，着力打造幸福家园。最后一章是强化规划实施，实现宏伟蓝图。总的看，《纲要(草案)》充分体现中央“四个全面”战略布局，严格遵循“五大发展”理念，具体落实市委“六个攻坚战”要求，确保了《纲要(草案)》适度超前、务实管用，具有很强的指导性和可操作性。

　　各位代表！

　　四平已经迈上了新的历史征程，任务繁重，前景光明。让我们在市委的坚强领导下，万众一心、攻坚克难，全民行动、求富图强，以更大的决心和勇气全面推进改革发展，为实现“美丽四平、幸福家园”建设目标奋勇前进!
